# 7,8-Dihydroxyflavone, a Small Molecule TrkB Agonist, Improves Spatial Memory and Increases Thin Spine Density in a Mouse Model of Alzheimer Disease-Like Neuronal Loss

**DOI:** 10.1371/journal.pone.0091453

**Published:** 2014-03-10

**Authors:** Nicholas A. Castello, Michael H. Nguyen, Jenny D. Tran, David Cheng, Kim N. Green, Frank M. LaFerla

**Affiliations:** 1 Institute for Memory Impairments and Neurological Disorders, University of California Irvine, Irvine, California, United States of America; 2 Department of Neurobiology and Behavior, University of California Irvine, Irvine, California, United States of America; The Florey Institute of Neuroscience and Mental Health, Australia

## Abstract

Augmenting BDNF/TrkB signaling has been demonstrated to be a promising strategy for reversing cognitive deficits in preclinical models of Alzheimer disease (AD). Although these studies highlight the potential of targeting BDNF/TrkB signaling, this strategy has not yet been tested in a model that develops the disease features that are most closely associated with cognitive decline in AD: severe synaptic and neuronal loss. In the present study, we investigated the impact of 7,8-dihydroxyflavone (DHF), a TrkB agonist, in CaM/Tet-DT_A_ mice, an inducible model of severe neuronal loss in the hippocampus and cortex. Systemic 7,8-DHF treatment significantly improved spatial memory in lesioned mice, as measured by water maze. Analysis of GFP-labeled neurons in CaM/Tet-DT_A_ mice revealed that 7,8-DHF induced a significant and selective increase in the density of thin spines in CA1 of lesioned mice, without affecting mushroom or stubby spines. These findings suggest chronic upregulation of TrkB signaling with 7,8-DHF may be an effective and practical strategy for improving function in AD, even after substantial neuronal loss has occurred.

## Introduction

The interaction between brain-derived neurotrophic factor (BDNF) and its high-affinity receptor, TrkB, initiates downstream signaling that is critical for plasticity and memory [1]–[4]. Recent studies have highlighted the potential of targeting BDNF/TrkB signaling for the treatment of neurodegenerative diseases such as Alzheimer disease (AD) [5]–[8]. Such work has demonstrated that TrkB activation improves cognition, and this improvement is associated with increased synapse density. Furthermore, the effects of TrkB activation on cognition are typically independent of any effect on Aβ or tau pathology [5], [6]. While promising, the efficacy of BDNF/TrkB-based therapies has yet to be demonstrated in a mouse model with severe neuronal and synaptic loss, such as occurs in AD [9], [10].

The loss of neurons is a prominent and early feature of AD, and this loss correlates with cognitive deficits more closely than amyloid load [9], [11], [12]. The hippocampal formation is particularly vulnerable, and in the late stages of the disease loss of neurons in the CA1 and layer II of the entorhinal cortex reaches 80–90% [13]. Even in mild AD, there is already a loss of over half of neurons in CA1 and layer II of the entorhinal cortex [10]. Patients can have significant plaque and tangle pathology without detectable cognitive impairment, and only once substantial neuronal loss develops do patients begin to show signs of dementia [10]. Since significant synaptic and neuronal loss has already occurred by the time symptoms manifest and AD is diagnosed, it may be useful to promote the restoration of lost synapses by targeting signaling pathways such as BDNF/TrkB.

Since BDNF has poor blood-brain barrier penetration and a short half life [14], TrkB has typically been targeted by locally upregulating BDNF through viral overexpression or direct protein infusion [6], [7], [15]. While these methods have provided a convincing proof of principle, other delivery methods should be developed to make clinical translation more feasible. Specific small molecule TrkB agonists have been recently developed that can be administered systemically and initiate TrkB activation with equal potency to BDNF [16], [17]. Treatment with these drugs has the benefit of being far less invasive than viral or recombinant protein delivery, and allows the chronic targeting of multiple affected brain regions.

One recently identified TrkB agonist, 7,8-dihydroxyflavone (DHF), has shown promising protective effects in mouse models of aging, Parkinson disease, and AD [8], [17]–[19]. In the current study, we investigate whether 7,8-DHF treatment can also reverse functional deficits related to severe neuronal loss using CaM/Tet-DT_A_ mice. The CaM/Tet-DT_A_ model harbors a tetracycline-inducible transgene system that selectively kills neurons of the hippocampus and cortex, and produces behavioral deficits in cognitive domains subserved by these regions [20]–[22]. The pattern and degree of neuronal loss in this model emulates that found in AD [9], [13], [23]–[25]. Furthermore, for the current study we modified the CaM/Tet-DT_A_ model to carry a Thy1-GFP-M transgene, which expresses GFP stochastically in CA1 pyramidal neurons [26] and allows analysis of the impact of 7,8-DHF on dendritic spine remodeling.

## Results

### Generating GFP-expressing CaM/Tet-DT_A_ Mice

**Figure 1:**
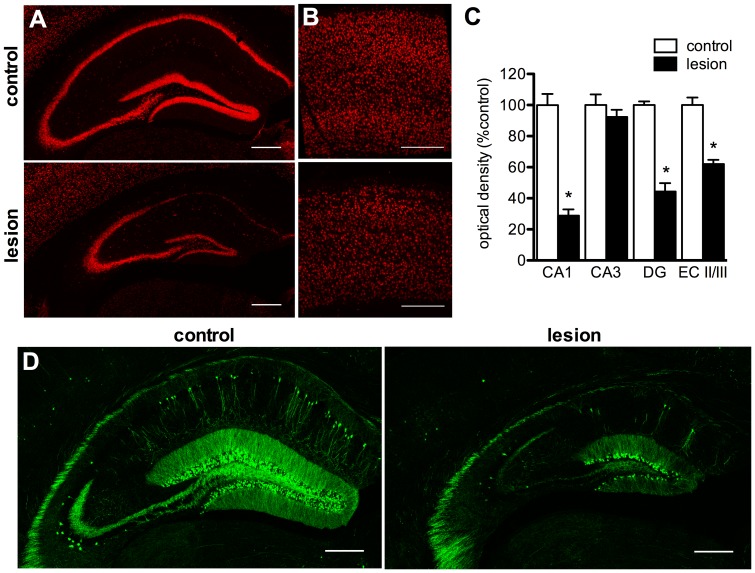
CaM/Tet-DT_A_ mice develop neuronal loss in Alzheimer-disease related brain areas. A 25-day withholding of doxycycline from the diet of CaM/Tet-DT_A_ mice induces DT_A_ expression, which causes a selective loss of hippocampal and cortical neurons. Representative images depict a significant loss of NeuN immunoreactivity in the CA1 and DG of the hippocampus (**A**), and layers II/III of the entorhinal cortex (**B**, *n* = 5–6, quantified by optical densitometry in **C**). Crossing CaM/Tet-DT_A_ mice with mice carrying a Thy1-GFP-M transgene produces sparse GFP-labeling of CA1 pyramidal neurons (**D**). Scale bars = 1 mm.

To facilitate the analysis of the impact of 7,8-DHF on dendritic spines, we modified CaM/Tet-DT_A_ mice to also carry a Thy1-GFP-M transgene, which produces a stochastic and sparse pattern of labeling in CA1 pyramidal neurons (Fig. 1D) [26]. Tet-DT_A_^+/+^ mice were bred with Thy1-GFP-M^+/−^, and subsequent generations were genotyped by Southern blot and interbred until animals that were homozygous for both transgenes were produced. Tet-DT_A_^+/+^/Thy1-GFP-M^+/+^ and CaM-tTA^+/−^ colonies were maintained separately and then crossed to produce CaM-tTA^+/−/^Tet-DT_A_^+/−/^Thy1-GFP-M^+/−^ or CaM-tTA^−/−/^Tet-DT_A_^+/−/^Thy1-GFP-M^+/−^ mice for experiments.

To ensure that the presence of GFP or new background strain elements did not disrupt the functioning of the CaM/Tet-DT_A_ lesion system, hippocampal and cortical lesions were induced in 3-month-old CaM-tTA^+/−/^Tet-DT_A_^+/−/^Thy1-GFP-M^+/−^ (CaM/Tet-DT_A_) mice by replacing mouse chow containing doxycycline, a tetracycline analog, with standard chow. Tet-DT_A_^+/−/^Thy1-GFP-M^+/−^ littermates were also taken off doxycycline chow, and since they do not carry CaM-tTA these animals served as nonlesioned controls. After 25 days, doxycycline was reintroduced to prevent further DT_A_ induction, and tissue was collected 45 days after lesion cessation.

To examine the extent of neuronal loss in GFP-expressing CaM/Tet-DT_A_ mice, we conducted immunohistochemistry for neuronal nuclei marker NeuN. Lesioned mice have 71% less NeuN immunoreactivity in the CA1 pyramidal layer compared to controls (Fig. 1A,C, *n* = 5–6, *p*<0.0001). This is highly similar to the 74% reduction in CA1 pyramidal neurons found in the characterization of the original, non-GFP-expressing CaM/Tet-DT_A_ mice [20]. This suggests the introduction of the Thy1-GFP transgene, and any new background strain gene variants, has not altered the functioning of the inducible lesion system. We also examined the extent of neuronal loss in other regions and found significant loss of NeuN immunoreactivity in the dentate gyrus granule cell layer (56%, Fig. 1A,C, *n* = 5–6, *p*<0.0001) and layers 2/3 of entorhinal cortex (38%, Fig. 1B–C, *n* = 5–6, *p*<0.0001). In contrast, we found no change in NeuN immunoreactivity in CA3 (Fig. 1A,C, *n* = 5–6, *p* = 0.38), which indicates these neurons are not affected by the lesion. We have found that CA3 pyramidal neurons do not express detectable levels of tTA (T. Yamasaki and F. LaFerla, unpublished findings), which precludes DT_A_ induction. As a result of the loss of these neuronal populations, there is overt reduction in hippocampal and cortical volume, which has been previously characterized (K. Myczek and F. LaFerla, unpublished findings).

The pattern of GFP labeling in CaM/Tet-DT_A_ mice is sparse within the CA1 (Fig. 1D), which is consistent with the original characterization of the Thy1-GFP-M mouse [26]. There are substantially fewer GFP-labeled neurons in the CA1, cortex, and DG of lesioned TGC mice, which indicates GFP-labeled neurons are vulnerable to DT_A_ induction.

### 7,8-DHF Treatment Improves Spatial Memory in CaM/Tet-DT_A_ Mice

To investigate whether treatment with 7,8-DHF improves spatial memory following severe synaptic loss, CaM/Tet-DT_A_ mice and Tet-DT_A_ mice were given daily i.p. injections of 7,8-DHF (5 mg/kg) or PBS for 2 weeks beginning 1 month after cessation of a 25 day DT_A_ induction. Mice were handled daily during the first week of treatment, and behavior testing began with the second week of treatment. Injections were given in the morning and handling/behavior was conducted approximately 5 hours later.

**Figure 2:**
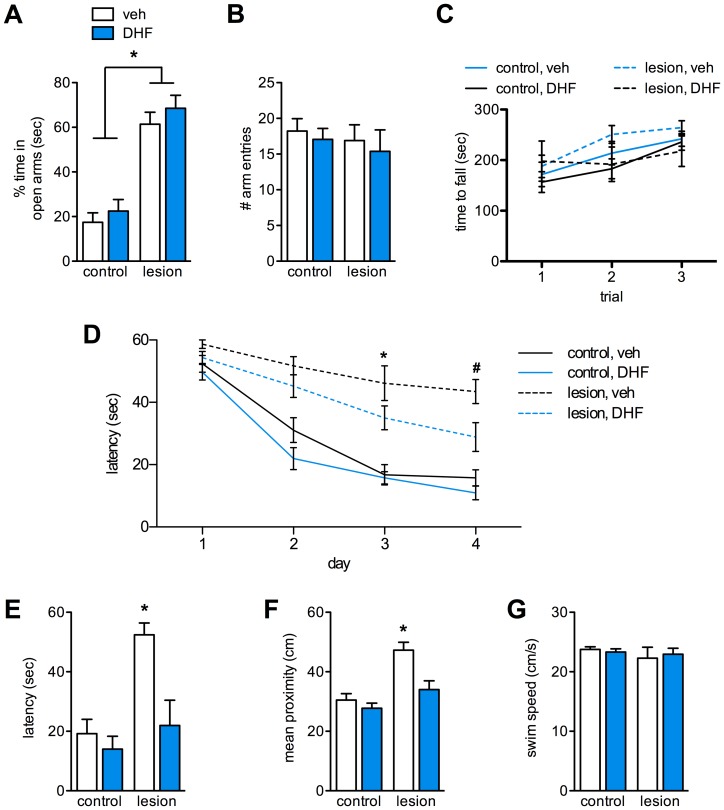
7,8-DHF treatment improves spatial learning and memory in CaM/Tet-DT_A_ mice. Lesioned CaM/Tet-DT_A_ mice exhibit a significantly increased preference for the open arms of an elevated plus maze compared to controls (**A**, *n* = 9–12, *p*<0.0001). All groups made a similar total number of arm entries (**B**). 7,8-DHF treatment did not alter preference for open or closed arms (**A–B**). A rotarod test reveals no differences between groups in the time to fall off an accelerating rod (**C**). During water maze training, lesioned mice treated with 7,8-DHF were not significantly slower to reach the escape platform compared to controls, except for day 3 (**D**, *n* = 9–12, *p*<0.001). On day 4, lesioned mice treated with 7,8-DHF reached the platform significantly faster than lesioned mice that received vehicle (**D**, *n* = 9–12, #, *p*<0.05). On the 24 hr probe trial, latencies to reach and mean proximity to the former platform location were not significantly different between 7,8-DHF-treated lesioned mice and controls (**E–F**, *n* = 9–12, *p*>0.05). All groups had a similar average swim speed during the probe trial (**G**).

To examine any impact of 7,8-DHF treatment on anxiety-like behavior, mice were tested on an elevated plus maze. Control mice spent 17% of the 5 minute trial in the open arms of the maze, whereas lesioned mice spent 61% of the time in open arms (Fig. 2A, *n* = 9–12, *p*<0.0001), although the groups explored the maze equally (Fig. 2B). 7,8-DHF had no impact on the percentage of time spent in the open arms of the maze for control or lesioned mice, and all groups explored the maze equivalently (Fig. 2A–B). To determine if the lesion affects motor coordination, we tested animals on the rotarod using a 3-trial accelerating protocol. All groups stayed on the rotarod for an equivalent length of time (Fig. 2C), indicating the lesion does not cause overt motor deficits.

To examine the impact of chronic TrkB agonism on hippocampus-dependent behavior, animals were tested on a water maze. Vehicle-treated lesioned mice were significantly slower than control groups to reach the hidden platform on days 2–4 of water maze training (Fig. 2D, *n* = 9–12, *p*<0.001). Lesioned mice treated with 7,8-DHF reached the platform significantly faster than vehicle-treated control mice on day 4 of water maze testing (Fig. 2D, *n* = 9–12, *p*<0.05). On days 2 and 4, escape latencies for 7,8-DHF-treated lesioned mice were not significantly different from vehicle-treated controls (*n* = 9–12, *p*>0.05). 24 hours after the last training day, mice were tested again on a 1-minute probe trial, in which the escape platform was removed. Lesioned mice were significantly slower than controls to reach the former platform location (Fig. 2E, *n* = 9–12, *p*<0.001) and were, on average, significantly further from the former platform location (Fig. 2F, *n* = 9–12, *p*<0.001). Probe latency for 7,8-DHF-treated lesioned mice was significantly lower than vehicle-treated lesioned mice (*n* = 9–12, *p*<0.01, Fig. 2E), and was not significantly different than vehicle-treated controls (*n* = 9–12, *p*>0.05). In addition, 7,8-DHF-treated lesioned mice were, on average, closer in proximity to the former platform location than vehicle-treated lesioned mice (Fig. 2F, *n* = 9–12, *p*<0.05), and this distance was equivalent to nonlesioned controls (*n* = 9–12, *p*>0.05). To ensure there were no differences in swimming ability or motivation, swim speed was measured by video tracking software and found to be equivalent for all groups (Fig. 2G). Each animal was sacrificed by Euthasol overdose and cardiac perfusion immediately following its probe trial, which took place approximately 5 hours after the final treatment injection.

### 7,8-DHF Treatment Increases Spine Density on Dendrites in CA1 Pyramidal Neurons

**Figure 3:**
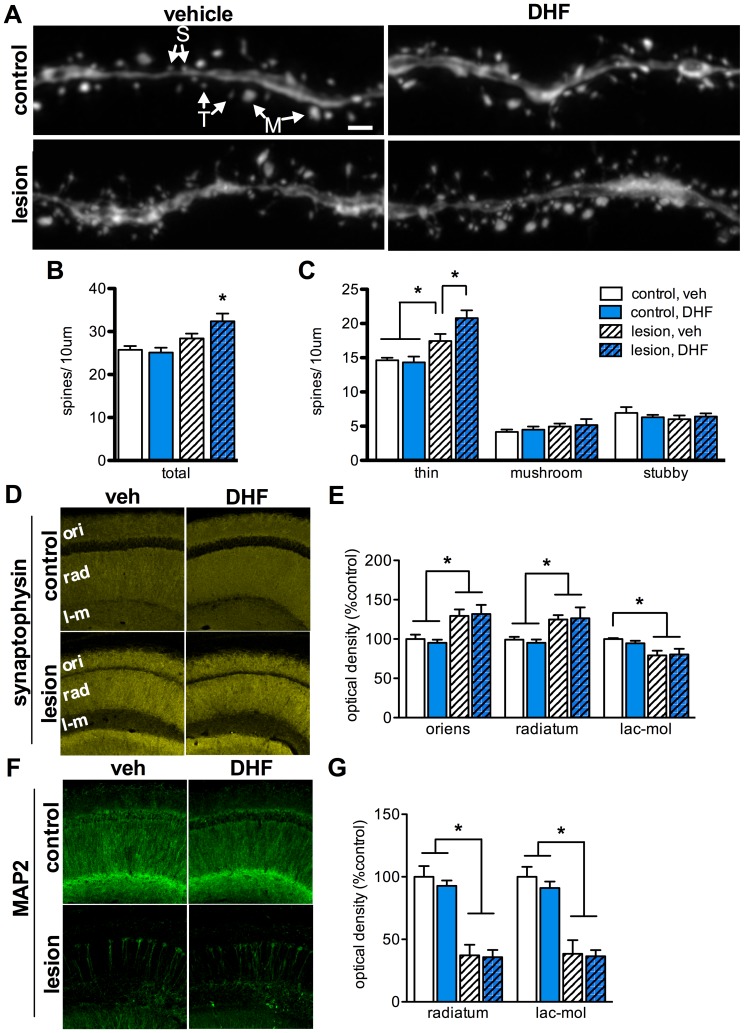
7,8-DHF treatment increases thin spine density on dendrites in CA1 pyramidal neurons. High-magnification confocal imaging and analysis of dendrites from GFP-expressing CA1 pyramidal neurons reveals elevated total spine density in 7,8-DHF-treated lesioned mice (**A–B**, *n* = 5–7, *p*<0.05). Classification of thin (T), mushroom (M), and stubby (S) spines indicates a specific elevation of thin spines in lesioned mice (**C**, *n* = 5–7, *p*<0.05), which is further elevated by 7,8-DHF treatment (*n* = 5–7, *p*<0.01 versus vehicle-treated lesioned mice). Analysis of synaptophysin immunoreactivity reveals a lesion-induced increase in presynaptic terminal packing density in CA1 stratum oriens (ori) and radiatum (rad) (**D–E**, *n* = 5, *p*<0.05), and loss of density in the stratum lacunosum-moleculare (**D–E**, *n* = 5, *p*<0.01), but no effect of 7,8-DHF. Labeling for MAP2 reveals a significant loss of CA1 pyramidal neuron dendrites in lesioned groups (**F–G**, *n* = 5–7, *p*<0.001), and no impact of 7,8-DHF treatment on dendrite density. Scale bar = 1 µm.

To determine if 7,8-DHF treatment alters spine density and morphology, we counted and classified spines on CA1 pyramidal neurons. Randomly selected GFP-labeled dendrite segments from CA1 stratum radiatum were imaged by confocal microscopy and a blinded investigator counted and classified dendritic spines according to their morphology. Spines were manually counted and classified as thin, mushroom, or stubby, according to previously described criteria (Fig. 3A) [27]. To ensure counts were accurate and included spines oriented parallel to the z-axis, counts were performed on intact (i.e., not collapsed) z-stacks by scrolling through the individual images of the stack. For each animal, approximately 100 µm of total dendrite length and 250–400 spines were analyzed. On average, vehicle-treated lesioned mice had a 10% greater total number of spines per length of dendrite compared to controls, although this difference did not reach significance (Fig. 3A–B, *n* = 5–7, *p* = 0.11). Lesioned mice treated with 7,8-DHF had significantly elevated total spine density versus nonlesioned controls (*n* = 5–7, *p*<0.05), and trended toward having higher total spine density than vehicle-treated lesioned mice. Analysis of spine density by morphology revealed a 19% higher density of thin spines in vehicle-treated lesioned mice compared to control mice (*n* = 5–7, *p*<0.05), but no difference in numbers of mushroom or stubby spines (Fig. 3C). 7,8-DHF treatment in lesioned mice resulted in a further elevation of thin spine density, which was significantly higher than nonlesioned controls (*n* = 5–7, *p*<0.001) and vehicle-treated lesioned mice (*n* = 5–7, *p*<0.01), again with no impact on the density of mushroom or stubby spines (Fig. 3C).

To examine the impact of 7,8-DHF on presynaptic terminal packing density we conducted an immunohistochemical analysis of synaptophysin in layers of CA1. Lesioned groups had significantly elevated synaptophysin immunoreactivity versus control groups in strata oriens (Fig. 3D–E, *n* = 5, *p*<0.05) and radiatum (*n* = 5, *p*<0.05). Since strata oriens and radiatum receive major input from Schaffer collaterals emanating from CA3 pyramidal neurons, which are unaffected by the lesion, this may be the result of the compaction of presynaptic terminals as hippocampal volume decreases. In contrast, presynaptic terminal density was decreased in stratum lacunosum-moleculare of lesioned animals relative to controls (Fig. 3D–E, *n* = 5, *p*<0.01). Since the entorhinal cortex is a major input into the stratum lacunosum-moleculare, the loss of presynaptic terminals in this layer is likely related to the loss of entorhinal cortex neurons. 7,8-DHF treatment did not alter synaptophysin density in control or lesioned mice for any layer of CA1 (Fig. 3D–E).

TrkB signaling can promote dendritic development and arborization [28], [29], so we examined the impact of 7,8-DHF treatment on CA1 dendrite density. In lesioned mice, too few GFP-expressing neurons remained to allow tracing and Sholl analysis of dendritic arbors. Instead, we analyzed immunoreactivity for MAP2, a microtubule stabilizing protein expressed in dendrites, which has expression that varies with dendritic arbor alterations [30]. Lesioned groups had significantly less MAP2 immunoreactivity in strata radiatum and lacunosum-moleculare (Fig. 3F–G, *n* = 5–7, *p*<0.001), indicating that the dendrites of killed pyramidal neurons may have been cleared. 7,8-DHF treatment did not alter MAP2 staining in control or lesioned groups, which suggests 7,8-DHF does not influence dendrite density. MAP2 was not analyzed for stratum oriens because expression there was very low for all groups.

## Discussion

Our studies indicate that chronic administration of a TrkB agonist, 7,8-DHF, significantly improves hippocampus-dependent behavior in the CaM/Tet-DT_A_ model of AD-like neuronal loss. Furthermore, we found that 7,8-DHF treatment in lesioned mice induced an increase in the density of dendritic spines, which may underlie the improvements in cognition. Interestingly, 7,8-DHF did not alter spine density in nonlesioned controls, indicating that TrkB signaling interacts with other signals initiated by the lesion to facilitate spine remodeling. Analysis of spine density by morphology revealed a selective increase in the density of thin spines, which are thought to be important for establishing nascent synapses following LTP and learning [31]. This selective increase in thin spines is consistent with previous studies that demonstrate rapid spine turnover and increased average spine length following neuronal loss by stroke [32], [33]. Thin spines are structurally flexible and are able to compartmentalize molecules like Ca^2+^ to modulate synaptic efficacy, which may make thin spines particularly suited for the formation of new synapses [34], [35]. These properties may also make them well suited for supporting the restoration of connectivity and function after injury.

Separate from the impact of 7,8-DHF, our findings also indicate the lesion induces a selective increase in thin spines, which may be indicative of an innate compensatory response. Previous work has demonstrated that synaptogenesis occurs in animals following experimental lesion or injury [36], [37]. This may be an attempt by the surviving neurons to compensate for lost connectivity, and in some lesion models these kinds of structural changes are associated with improved function [38], [39]. Although lesioned CaM/Tet-DT_A_ mice still have severe functional impairments, thin spine density may be elevated in an attempt to support the formation of new, compensatory synapses with innervating fibers.

Previous studies have also found that augmenting TrkB signaling leads to increased spine density [40], [41]. It is unclear, however, whether TrkB signaling promotes the addition of new spines, or slows the elimination of spines that normally undergo rapid turnover. Under some conditions, TrkB activation appears to be critical for the maintenance of spine density [42]. Since the lesion alone leads to a significant increase in thin spine density, it is possible that enhancing TrkB signaling with 7,8-DHF promotes the stabilization of spines that would have been eliminated. Further studies are needed to determine whether these thin spines persist, but if restoration after injury resembles development we might expect that over time many of these spines will be eliminated, and a subset of thin spines will mature into larger and more stable, mushroom-like spines [35].

TrkB activation has been previously demonstrated to promote axon spouting [5], [43], [44], however our findings suggest 7,8-DHF had no impact on presynaptic terminal packing density. While presynaptic terminal remodeling likely occurs to re-establish synapses with nascent spines, our data suggests there is no change in the total number axon terminals. For CA1 strata oriens and radiatum, which receive major innervation from the intact CA3, there is probably already an excess of presynaptic terminals relative to the number of spines.

Numerous recent studies have highlighted the therapeutic potential of targeting TrkB for recovering functional deficits related to neurodegenerative disease and injury [5]–[8], [45]–[47]. Our current findings expand upon this body of work and highlight the potential of targeting TrkB to reverse cognitive deficits caused by severe neuronal loss, a feature that is absent in many models of neurodegenerative disease. More generally, our findings suggest that even severely damaged networks of neurons are capable of undergoing structural remodeling that leads to the restoration of function.

## Materials and Methods

### Animals

All procedures were specifically approved by the Institutional Animal Care and Use Committee of the University of California, Irvine (protocol number: 1999–1706). All procedures were performed with strict adherence to this protocol and all efforts were made to minimize suffering. GFP-expressing CaM/Tet-DT_A_ mice were produced by crossing female, homozygous Tet-DT_A_ mice [20] with a hemizygous Thy1-GFP-M male (Jackson Labs, Bar Harbor, ME, catalog #007788) and interbreeding offspring until homozygosity was reached for both transgenes. The genotype of each animal of each generation was determined for Tet-DT_A_ and Thy1-GFP by Southern blot, as described below. Homozygous Tet-DT_A_/Thy1-GFP mice were maintained by crossing homozygotes, and then crossed to hemizygous CaMKIIα-tTA mice to produce CaM^−/−/^Tet-DT_A_^+/+^/GFP^+/+^ and CaM^+/−/^Tet-DT_A_^+/+^/GFP^+/+^ mice for experiments, herein referred to as control and lesioned, respectively. CaMKIIα-tTA genotype was determined by PCR with the following primers: 5′-CGCATTAGAGCTGCTTAATG-3′ and 5′-TCGCGATGACTTAGTAAAGC-3′. To suppress induction of DT_A_, mice were maintained on a diet supplemented with 2000 parts per gram doxycycline (Research Diets, New Brunswick, NJ). This diet was replaced with normal mouse chow for 25 days to induce diphtheria toxin A-chain (DT_A_) expression. To abrogate DT_A_ expression, doxycycline chow was replaced and mice were also given doxycycline-supplemented drinking water (2 mg/ml, Sigma-Aldrich) for 2 days. After 2 days, mice were given regular drinking water but remained on doxycycline chow.

### Southern Blot Genotyping

Template DNA for Tet-DT_A_ was generated by growing bacteria carrying a tetracycline responsive element (Tet)-containing plasmid, digesting with Xba1 and BamH1, and then gel extracting the target fragment. To generate GFP template, tail DNA was isolated from a known Thy1-GFP-M hemizygote, a 701 bp fragment of eGFP was amplified by PCR (primers: 5′-CAAGGGCGAGGAGCTGTTCACC-3′ and 5′-GCTCGTCCATGCCGAGAGTGATC-3′), and then the fragment was gel extracted. 10 µg of genomic DNA isolated from the tail tip of each animal was restriction digested overnight with either EcoRV (Thy1-GFP) or SacI (Tet-DT_A_) (New England Biolabs, Ipswich, MA). Electrophoresis-separated genomic DNA was transferred to a nitrocellulose membrane by capillary action and then crosslinked by UV irradiation. Hybridization probe was generated by incubating template DTA with ^32^P-dCTP for 1 hour with labeling beads (GE Healthcare). DNA membranes were incubated in hybridization solution overnight at 55°C and then exposed to film.

### Behavioral Procedures

All mice were 3.5–5-months-old at the start of behavior testing, and groups were composed of age- and sex-matched mice from at least 4 independent litters. Littermates were assigned to separate groups to avoid litter effects. Animals were tested for anxiety-like behavior on an elevated plus maze, based on a previously described protocol [48]. The elevated plus maze consists of 4 intersecting arms (5×30 cm), two of which have walls (“closed”, 15 cm in height) and the other two of which have no walls (“open”). The entire maze is elevated 40 cm above the ground and placed in the center of a room with lighting adjusted to 15 lux. Animals were placed at the junction of these arms and allowed to freely explore the maze for 5 min. Video footage was captured and later analyzed by a blinded investigator for the time spent in open and closed arms, and the total number of arm entries. An animal was considered in an arm whenever the body (not including the tail) was entirely in the arm. The maze was thoroughly cleaned with 70% ethanol in between trials.

Animals underwent rotarod testing of motor deficits based on a previously described protocol [49]. Animals were tested on a rotarod apparatus (Ugo Basile, Italy) for 3 trials, separated by 10 min. For each trial, the rotarod accelerated linearly from 4 to 40 rpm over a 5 min period. The trial stopped for each animal once it fell off the rotarod or held onto the rotarod without walking for 2 consecutive revolutions. The maze was cleaned with Virkon in between trials.

Water maze testing was conducted according to previously described procedures [50]. Briefly, a 1 m diameter circular pool filled with opaque water maintained at 29°C. During training, mice were placed into the pool and allowed to find and climb onto a submerged 12 cm diameter platform for 4 trials per day. After 4 days of training, the escape platform was removed and mice were tested 24 h later on a probe trial. The probe trial was videotaped and later analyzed with EthoVision XT (Noldus, Wageningen, The Netherlands). The training phase was analyzed by repeated measures ANOVA with Bonferroni posthoc tests. Probe trial measures were analyzed by 1-way ANOVA with Bonferroni posthoc tests.

### Tissue Processing

Mice were deeply anesthetized with sodium pentobarbital and then transcardially perfused at a rate of 11 ml/min with cold PBS. Brains were removed and fixed in 4% paraformaldehyde for 48 hours and then cryoprotected in 30% sucrose. Cryoprotected brains were then frozen on dry ice and sectioned coronally at 40 µm using a sliding microtome (Leica Microsystems, Richmond, IL). Sections were collected into PBS with 0.02% sodium azide and stored at 4°C.

### Immunohistochemistry

A representative subset of the tissue collected from the behavior experiments was randomly selected for histological analysis. Free-floating sections from 1.7 to 2.3 mm posterior to Bregma were washed in PBS, and then incubated in block (PBS with 0.2% Triton X-100, 3% BSA, and 3% normal goat serum) for 1 hr. Sections were incubated overnight at 4°C in primary antibodies for MAP2 (1∶2000, Millipore), NeuN (1∶1000, Millipore), or synaptophysin (1∶3000, Sigma) diluted in block followed by 1 hr incubation at room temperature in Alexa Fluor 555 fluorescent secondary antibody (1∶200, Life Technologies, Grand Island, NY). Finally, sections were mounted onto slides and coverslipped in Vectashield mounting media (Vector Laboratories, Burlingame, CA). Immunofluorescently labeled sections were imaged with a Leica DM2500 TCS SPE laser scanning confocal microscope. Identical scan settings were used for all samples for each brain region analyzed. Z-stacks were collapsed into a maximum intensity projection and analyzed for mean pixel intensity.

### Dendritic Spine Analysis

A single section from 1.7 to 2.3 mm posterior to Bregma was selected for each animal, mounted onto slides, and coverslipped. For each animal, 5 non-primary, apical dendrite segments in CA1 stratum radiatum were selected at random by a blinded investigator. Z-stacks of dendrite segments were acquired at 100× with 4× zoom and a 0.15 µm step size using a Zeiss LSM780 laser scanning confocal microscope, and then deconvoluted using Huygens Professional (Scientific Volume Imaging, The Netherlands). Deconvoluted stacks were analyzed with ImageJ (NIH) using the Cell Counter feature to manually count and classify dendritic spines based on previously used criteria [27]. Briefly, spines were considered thin if the neck was much smaller than the length, but similar to the head diameter. Mushroom spines have a very large head diameter which tapers to a much thinner neck. Stubby spines have a neck diameter that is roughly equivalent to the spine length, such that it appears “neckless”. Stacks were then collapsed into a maximum intensity projection and dendrite segment length was using the freehand tracing tool in ImageJ.

### Statistical Analyses

All data are expressed as the mean ±SEM. Data were analyzed using planned 1-way ANOVAs with Bonferroni posthoc tests. Data were analyzed using GraphPad Prism 5 (San Diego, CA) and results were considered significant if *p*<0.05.
